# *ZebraBeat*: a flexible platform for the analysis of the cardiac rate in zebrafish embryos

**DOI:** 10.1038/srep04898

**Published:** 2014-05-09

**Authors:** Elisa De Luca, Gian Maria Zaccaria, Marwa Hadhoud, Giovanna Rizzo, Raffaele Ponzini, Umberto Morbiducci, Massimo Mattia Santoro

**Affiliations:** 1 Department of Molecular Biotechnology and Health Sciences and Molecular Biotechnology Center, University of Torino, Turin, Italy; 2 Department of Mechanical and Aerospace Engineering, Politecnico di Torino, Turin, Italy; 3 Faculty of Engineering, Helwan University, Helwan, Egypt; 4 Institute of Bioimaging and Molecular Physiology, CNR, Milan, Italy; 5 SuperComputing Applications and Innovation Department, CINECA, Milan, Italy; 6 Vesalius Research Center, VIB, KU Leuven, Leuven, Belgium

## Abstract

Heartbeat measurement is important in assesssing cardiac function because variations in heart rhythm can be the cause as well as an effect of hidden pathological heart conditions. Zebrafish (*Danio rerio*) has emerged as one of the most useful model organisms for cardiac research. Indeed, the zebrafish heart is easily accessible for optical analyses without conducting invasive procedures and shows anatomical similarity to the human heart. In this study, we present a non-invasive, simple, cost-effective process to quantify the heartbeat in embryonic zebrafish. To achieve reproducibility, high throughput and flexibility (i.e., adaptability to any existing confocal microscope system and with a user-friendly interface that can be easily used by researchers), we implemented this method within a software program. We show here that this platform, called *ZebraBeat*, can successfully detect heart rate variations in embryonic zebrafish at various developmental stages, and it can record cardiac rate fluctuations induced by factors such as temperature and genetic- and chemical-induced alterations. Applications of this methodology may include the screening of chemical libraries affecting heart rhythm and the identification of heart rhythm variations in mutants from large-scale forward genetic screens.

The zebrafish (*D. rerio*) is a model organism that has rapidly reached a respected position in biomedical research^1^,^2^. The zebrafish has traditionally been used as a model for developmental biology because of its many advantages, such as its rapid external development, the large number of eggs produced by a single cross, and the ease of genetic manipulation^3^,^4^. Another important feature of the embryonic zebrafish is its optical clarity, which overcomes the problem of limited accessibility of organs in other model organisms. The optical clarity of zebrafish embryos and larvae throughout embryogenesis allows the real time, *in vivo* observation of physiological or pathological events that occur during organ development. In this regard, the zebrafish is an optimal vertebrate model system for studying cardiovascular development and homeostasis^5^,^6^. In fact, the zebrafish has a relatively simple cardiac and vascular system, and the molecular mechanisms underlying vessel formation and morphogenesis are very similar to those of higher vertebrates, showing a remarkable degree of anatomical and functional conservation^7^.

The accessibility of internal organs in the zebrafish, including the heart, the blood and the blood vessels, makes it an ideal candidate for the study of the mechanisms underlying cardiovascular diseases^8^,^9^,^10^,^11^,^12^ using *in vivo* non-invasive imaging techniques. The heart is the first organ to form in zebrafish. At early stages of development (24 hpf), it is a linear tube that undergoes a process of remodeling (looping), which ends in the formation of two chambers (a single atrium and a single ventricle), separated by the atrio-ventricular valve, which prevents blood backflow, and the outflow tract (*bulbous arteriosus*)^13^,^14^. Additionally, because zebrafish embryos can take in oxygen from water by passive diffusion, malformations in the zebrafish cardiac system that lead to the absence of blood circulation are compatible with embryonic development, growth, and survival, allowing easy investigation of the causes of such dysfunctions^15^.

Among the parameters for cardiac function that can be studied in embryonic zebrafish, heart rate variability are of the utmost importance. Because these parameters are altered with cardio-toxicity in human beings, embryonic zebrafish are useful models for revealing the cardio-toxic and neurotoxic effects of pharmacological compounds in drug discovery. Moreover, these parameters can be fruitfully used to study developmental aspects of the sympatho-vagal balance in the cardiovascular system^16^. A number of methods have been developed to detect and quantify heart rate in zebrafish, including visual inspection, the application of *ad hoc* electrocardiographic devices and methods based on image processing^16^,^17^,^18^,^19^. Although evaluation of the cardiac rate by visual inspection is operator dependent and time consuming, the advantage of recording electrocardiographic signals is that clearly distinguished cardiac events can be easily observed in ECG traces. However, recording electrocardiographic traces from an embryonic zebrafish needs precise positioning of the electrodes, a crucial step for obtaining reproducible signals^17^,^18^.

The extensive use of time lapse imaging has allowed the recording of dynamic processes, such as blood flow and heart contraction, in transgenic zebrafish lines^20^. In particular, the introduction of fast recording tools such as confocal scanners has represented progress for image-based methods devoted for evaluating embryonic cardiac rate. For example, confocal laser-scanning microscopy has been used for quantitative measurement of cardiovascular performance in embryonic zebrafish^21^. High-speed video imaging has also been used to (1) determine heart rate variability and heart rhythm by studying blood cell velocity with digital motion analysis^16^,^22^,^23^ and to (2) measure heartbeat regularity through the acquisition of flowing blood images in caudal vasculature^19^. Although these methods are reliable and validated, they have not been integrated into a user-friendly- and freely available interface – e.g., involving software – for the many labs interested in using the zebrafish as model to monitor cardiac rate under different experimental conditions.

Here, we present a non-invasive approach that allows the fast, reliable and automatic assessment of cardiac rate in embryonic zebrafish. The proposed method allows the evaluation of heart rhythm in transgenic embryos from sequential images acquired with a resonant laser-scanning confocal microscope by (1) tracking the movement of the heart edges and determining the chronology of heart contraction/relaxation events (in terms of area variation) and/or (2) quantifying blood cell content in embryonic heart chambers during the cardiac cycle. The method is implemented in a prototype software called *ZebraBeat*, which has a user-friendly interface and allows reproducibility, flexibility and high throughput. In this study, the platform is successfully applied to a wide range of studies involving embryonic zebrafish: i.e., for monitoring cardiac rate in wild-type embryos at various developmental stages and in genetic mutants with defects that were chemically induced at early stages of heart development. An example of the strength of the proposed approach for further investigations, a study of the coupling/uncoupling of ventricular and atrial contraction, is also presented.

## Methods

### Zebrafish strains, maintenance and sample preparation

Zebrafish embryos were raised and kept under standard laboratory conditions at 28.5°C as previously described^24^. In experiments where drugs and morpholinos were tested, we used the double transgenic line *Tg(kdrl:GFP)^s843^;Tg(gata1:DsRED)^sd2^*, which expresses green fluorescent protein (GFP) in endocardial and endothelial cells^25^ and red fluorescent protein (DsRED) in red blood cells^26^. The *nemo (nem)^s838^* mutant line, identified from ENU-based mutagenesis screening as previously reported^27^, was selected to perform the analysis in mutant embryos. For embryonic heart visualization and image acquisition, embryonic zebrafish were anesthetized with tricaine and placed in 96-well plates (Ibidi, Cat. No. 89621), embedded in E3 medium containing N-phenylthiourea (PTU) to inhibit pigmentation.

### Ethics statement

Experimental procedures related to fish manipulation followed previously reported recommendations^28^ and conformed with the Italian regulations for protecting animals used in research, including DL 116/92.

The Ethics committee of the University of Torino approved this study. Larvae were anesthetized and, then, sacrifice by ice chilling.

### Image acquisition system

Image acquisition was performed using an automated Leica TCS SP5X II confocal laser-scanning microscope equipped with a tandem scanning system (Leica Microsystems, Wetzlar, Germany) with a dry objective HC PL FLUOTAR 20X (NA 0.5). RGB images were captured in the bidirectional mode (scanning frequency of 8000 Hz), at a frame resolution of 500 × 200 pixels. The acquired images were stored in three different channels: the green image, acquired with the 488 nm laser line; the red image, acquired with the 561 nm laser; and the gray-scaled image (i.e., the phase domain). After identification of a region of interest (ROI) containing the fluorescent signal and related tissues, images were acquired at minimum time intervals of 15 ms, for a total duration of 4.54 s (304 frames; sampling frequency of 67 frames per second). Each recorded frame was stored in TIFF format for image analysis.

### Method development, image processing and heart rate measurement

Two different image-based strategies were applied to evaluate the cardiac rate of the embryonic zebrafish. The first one was based on the assessment of the time-varying anatomy of the embryonic heart, and the second one was based on the assessment of the time-varying presence of blood cells in the heart chamber during the cardiac cycle.

For the first strategy (mode 1), a fully automated segmentation strategy was applied to each acquired image in order to identify the time-varying anatomy of the heart chamber. This task was completed by implementing a dynamic deformable model over the green channel images, where GFP in the endocardium is recorded. To avoid manual intervention by expert operators for defining a starting deformable model in the dataset and to speed up the process, a pre-processing strategy was established as follows. First, green channel images were selected and converted into gray-scale images. Second, gray-scale images were binarized in a two-step process: (1) the local minimum of the image histogram of the distribution of intensities was obtained, and this value was considered to be a threshold value; (2) the gray-scale image was converted to a binary image by using the threshold identified in step (1). As a final pre-processing step, a sequence of dilation/erosion-based morphological operators with 3 × 3 structuring elements was applied to filter the binary image. The highly connected binary images obtained in the pre-processing step allowed us to perform segmentation by applying a simple scheme. The segmentation of each binary image was obtained by tracing the outline of the “object” ventricle. In each binary image, zero pixels belong to an object (i.e., the embryo's heart chamber), and non-zero pixels constitute the background. Technically, we automatically defined a two-element vector **M**, specifying the row and column coordinates of the point on the object boundary where the tracing of the border to begin was defined. The search direction for the next object pixel connected to **Μ** was specified by rows and then by columns. After detecting the border of the endocardium at each time frame, the identified border was applied as a mask to the binary image and used to calculate the enclosed surface area (in terms of the number of image pixels). Thus, it was possible to track the movement of the heart edges and to extract the time series of the surface area variation across several cardiac cycles, a representative duration of heart contraction/relaxation events.

The second image-based strategy (mode 2) was based on the automatic processing of red channel images. Considering that an 8-bit image intensity color range is 0–255 and that the value of pixels located in non-moving regions (i.e., regions in the image where no red-colored blood cells are present) is equal to zero, the number of over-threshold pixels, representing pixels occupied by blood cells, was counted and expressed as the percentage of the total number of pixels in each frame. A threshold value of 50 in pixel intensity was set to reduce noisy artifacts. This threshold value was identified after a sensitivity analysis performed over a wide subset of acquired embryo images (by construction of a cumulative histogram of pixel intensities). Additionally, for this second approach, after detecting the percentage value of pixels representative of blood cells at each time frame, the time series of the blood cell number variation across several cardiac cycles was established. The time series determined by applying both strategies was transformed into a frequency spectrum using the Fast Fourier Transform (FFT) algorithm for the calculation of the power spectral density (PSD). The dominant frequency in PSD corresponded to the heart rhythm.

### Software development and functionality

A user-friendly, automated and high-throughput software program was developed within the MATLAB environment. The level of automation of the software requires two simple actions (mouse clicks) by the operator: (1) the selection of the main folder containing images to be analyzed (for each embryo, acquired images are automatically stored in a subfolder); and (2) the choice of analyzing the acquisition of a single embryo (by selecting the specific subfolder of the embryo of interest) or the whole pool of acquired embryos that were present in the multiwell plate (there is no upper limit for this number).

At the end of the processing step, if the operator makes the choice to analyze images from a single embryo, the results are immediately made available. However, if the operator makes the choice to analyze all of the acquired embryos, a report is generated containing the cardiac rate of all embryos and their identifiers. Finally, the operator can visualize the time history of the area variation and/or blood pool content or at the frequency spectrum of a specific embryo of the analyzed pool by simply loading the subfolder containing the analyzed images of the embryo of interest. The graphical user interface, as well as the operational mode, is illustrated in Supplementary Figures S1 and S2. This software is available to the scientific community upon request.

### Drug and pharmacological treatments

*Tg(kdrl:GFP)^s843^;Tg(gata1:DsRED)^sd2^* embryos at 96 hpf were arrayed in 96-well plates as previously described and maintained in E3 medium until drug treatment. The cardiac rate of each individual embryo was recorded immediately before the addition of each drug. Tricaine (MS-222, A5040, Sigma) or 2,3-BDM (2,3-butanedione monoxime, 31550, Sigma) was added in E3 medium at final concentrations of 500 μM and 20 μM, respectively. DL-Isoproterenol (isoprenaline hydrochloride, I5627, Sigma) and norepinephrine ((±)-Norepinephrine (+)-bitartrate salt, A0937, Sigma) were used at 50 μM and 1 mM, respectively.

For the analysis of the effects of pharmacological treatments on cardiac rate, embryos were treated with the following drugs: NS-398 (N194 Sigma) at 30 μM, L-NAME (L-NG-Nitroarginine methyl ester, Sigma) at 500 μM, SNAP (S-Nitroso-N-Acetyl-D,L-Penicillamine) at 100 μM or vehicle alone from 48 to 72 hpf. For PDE inhibition, 100 μM IBMX (I5879, Sigma), 100 μM caffeine (C0750, Sigma), 100 μM Ro-201724 (4-(3-Butoxy-4-methoxybenzyl)-2-imidazolidinone, 557502, Calbiochem), 50 μM Cilostamide (N-Cyclohexyl-N-methyl-4-(1,2-dihydro-2-oxo-6-quinolyloxy)butyramide (0915, Tocris) or vehicle was added from 48 to 72 hpf. After 24 hours of treatment, embryos were placed in 96-well plates for imaging and re-incubated with fresh drugs/chemicals for the duration of the experiments.

### Antisense morpholino oligonucleotide injections

Gene knockdown was performed by the microinjection of antisense morpholino oligonucleotides (Gene Tools, LLC) as previously described^29^: *gata1a*^ATG^ (5′-CTGCAAGTGTAGTATTGAAGATGTC-3′) and *gata2a*^Spl^ (5′-CATCTACTCACCAGTCTGCGCTTTG-3′)^30^. A standard morpholino (5′-CCTCTTACCTCAGTTACAATTTATA-3′) was used as the control. Morpholinos were injected into one-cell stage embryos at the following concentrations: 2.3 nl of 1 mM *control* MO, 2.3 nl of 1 mM *gata1a*^ATG^ MO, and 2.3 nl of 0.1 mM *gata2a*^Spl^ MO. The phenotype of the morphant embryos was assayed at 72 hpf.

### Statistical analysis

Statistical analysis was performed using Graph Pad Prism V6.0. Data are presented as the means ± SD. The differences between the means were tested for significance using the non-parametric Mann–Whitney U-test. A difference between two means was considered to be significant when p < 0.05 (*p < 0.05, **p < 0.01, ***p < 0.001).

## Results

### *ZebraBeat*, an image-based platform for measuring the cardiac rate in zebrafish embryos

**Figure 1:**
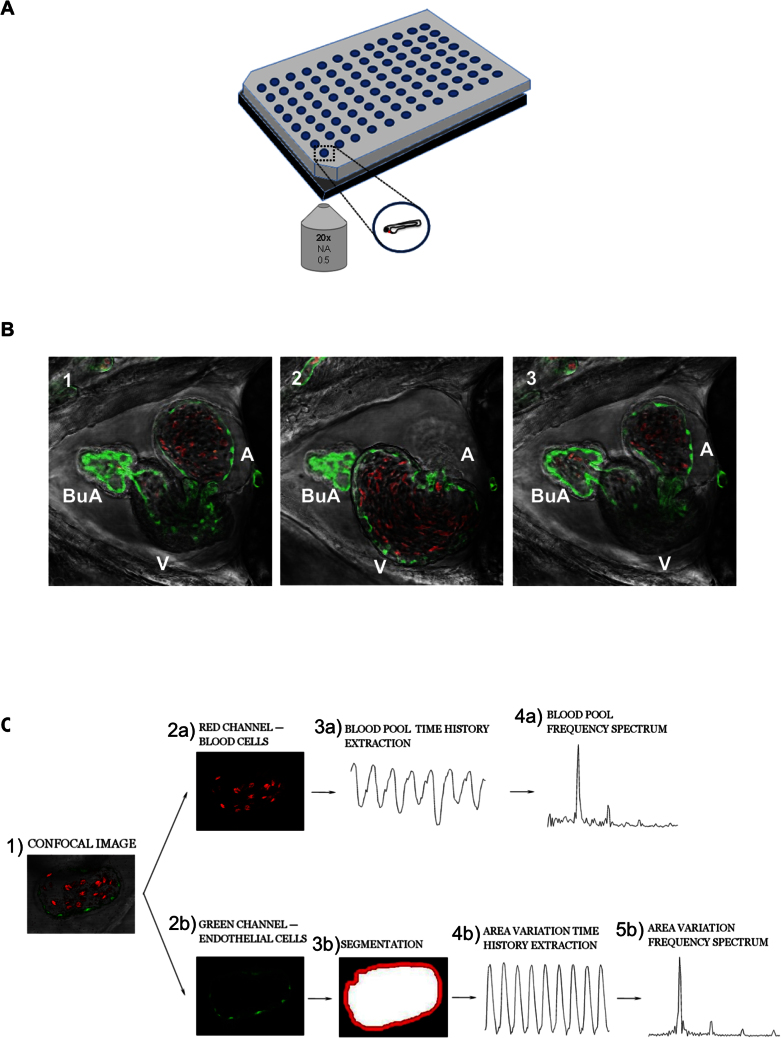
Measuring zebrafish heart rate using a novel image-based, automated method. (A) Illustration of sample preparation in 96 multiwell plates for confocal imaging and recording. (B) Three representative frames extracted from a time-lapse movie of a *Tg(Kdrl:GFP)^s843^/Tg(gata1:DsRED)^sd2^* embryo at 96 hpf. Images were acquired with a confocal resonant scanner. Merged bright-field/GFP/DsRED channel images of the *bulbous arteriosus*, (BuA), the ventricle (V), and the atrium (A) are shown. The complete movie is available as Supplementary Movie 2. (C) Schematic representation of the automated method in the two different “modes” of acquisition. According to the tissue analyzed, two different “modes” can be used. In mode 1 (area variation), GFP-positive heart wall tissue (endocardium) was selected (2b) and analyzed by segmentation of the area (3b). Subsequently, the chronology was determined (4b) from each frame and then converted into frequency (5b). In mode 2 (blood pool variation), DsRED-positive blood cells were analyzed (2a), and from the pixels at each time frame, the chronology (3a) was extracted and converted into frequency (4a). The two *modes* are interchangeable and can be used together depending on specific experimental conditions, such as the use of fluorescent transgenic animals or the lack of specific tissue (e.g., the loss of blood cells, which in zebrafish embryos is still compatible with cardiac development)^14^.

Embryonic zebrafish are characterized by an optical transparency that allows excellent visualization of internal organs and their functional movement, such as the heart beating and the blood flow within the heart chambers and vasculature. This unique feature is very useful for advanced stereomicroscopy in transgenic animals expressing fluorescent markers (GFP or DsRED) in cardiovascular tissues: for example, *Tg(Kdrl:GFP^s843^:gata1:DsRED^sd2^*) (Supplementary Movie S1). Therefore, detailed information on heart development, homeostasis and function can be obtained. Here we used this Tg line to analyze heart function in more detail, by exploiting confocal resonant scanner technology together with an automated image-processing method. Initially, we established a simple way of immobilizing live double fluorescent transgenic zebrafish embryos and imaged their hearts using a confocal resonant scanner (Figure 1A and 1B and Supplementary movie S2). In this study, we imaged the beating hearts of zebrafish embryonic with high-resolution, tracking the motion of the walls of the cardiac atrium (A), ventricle (V) and *bulbous arteriosus* (BuA) in different phases of the cardiac cycle in one channel (GFP, green). Additionally, using a second fluorescent channel (DsRED, red), we visualized blood cells streaming into the heart chambers. Thus, this system allows live automatic recording of the dynamic contraction of the zebrafish embryonic heart.

After acquisition of images, an automated processing method was applied for the assessment of the embryonic zebrafish cardiac rate. This method is illustrated in Figure 1C, and it is useful for rapidly and easily assessing the frequency of heart contraction under different experimental conditions. The basic steps of the method are as follows:

1. acquisition of images of the beating heart chamber using a confocal resonant scanner;
2. image segmentation for the identification of the cardiac chamber wall;
3. calculation of (a) the area enclosed within the segmented region at each time frame and extraction of the time history of the enclosed area, which varies according to the contraction and dilation of the heart chamber across cardiac cycles, or of (b) the blood pool content at each time frame, which varies according to the cardiac phase; and
4. extraction of the time history (of the blood pool content and/or the area variation of the segmented cardiac chamber) along with the cardiac phases.

**Figure 2:**
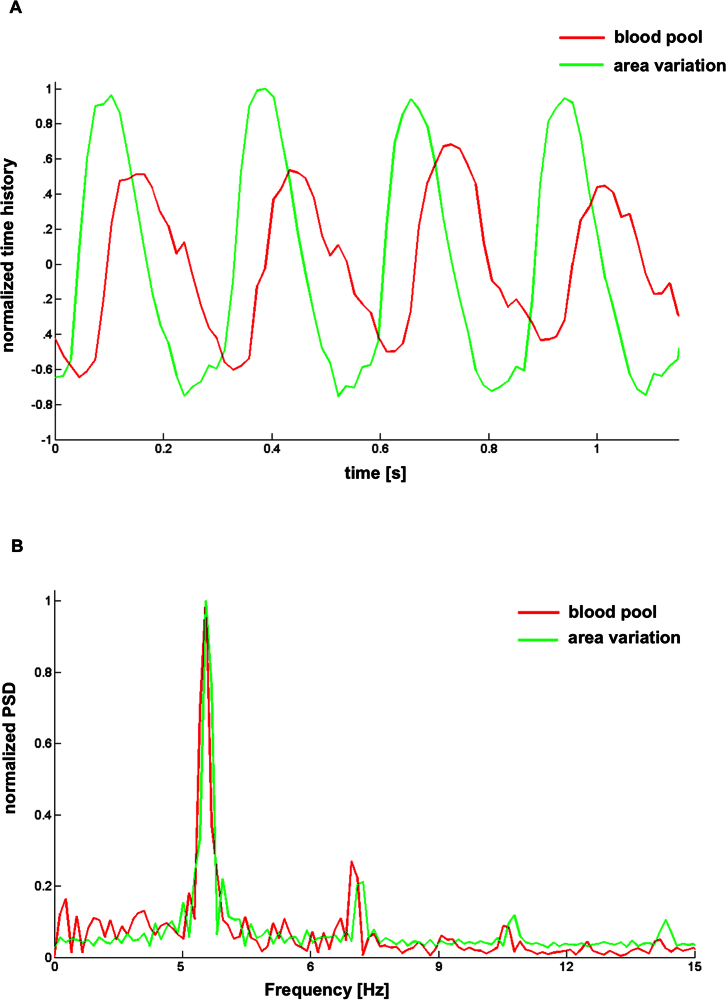
Chronologies of blood pool and area variation for four consecutive heart cycles of zebrafish embryos as determined based on recorded images. (A) Example of the blood pool and area variation across four cardiac cycles as extracted from a 96 hpf zebrafish *Tg(Kdrl:GFP)^s843^/Tg(gata1:DsRED)^sd2^* embryo. Images, analyzed both with mode 1 (green) and mode 2 (red), show similar results. (B) Frequency spectra of the chronologies as extracted from the green and the red channels of the RGB images, where the dominant frequency corresponds to the embryonic heart rhythm. The frequency spectrum was obtained using the Fast Fourier Transform method. The results are indicated as normalized PSD (power spectral density) and frequency (Hz). Images, analyzed both with mode 1 and mode 2, show (1) a small delay in peak occurrence in the blood pool chronology and (2) the same dominant frequency in the spectra.

As an example of what can be obtained as an output from the tool, Figure 2A presents the almost periodic time histories of the area and blood pool variation across four cardiac cycles of a 96 hpf wild-type embryo, as shown by the green and red channels of RGB images. The presence of a mild shift between peak values in the two time histories (area and blood pool) is observed, and the blood pool content is delayed with respect to the area variation of the heart chamber. By applying the FFT algorithm to the time histories, we can obtain the frequency spectra, from which we can easily identify the cardiac rhythm of the embryo under study, which corresponds to the dominant frequency of the spectrum (Figure 2B). As shown in the representative example in Figure 2, the application of the area variation and of the blood pool method leads to the identification of the same dominant frequency for the embryonic cardiac rate.

To achieve a user-friendly interface for measuring the heart rate of zebrafish hearts, we integrated this method into a software program that was developed in the MATLAB® environment. The graphical user interface and instructions on how to use the software are presented in Figure S1 and S2. We called the software *ZebraBeat*. A practical demonstration of how to use this software is presented in Supplementary Movie 3.

By combining the use of Tg zebrafish with cardiovascular-specific fluorescence, resonant scanner confocal microscopy, and image and signal processing, we developed a method and an associated software program that allow quantitative analysis of the cardiac rate of embryonic zebrafish. The effectiveness of the *ZebraBeat* platform at detecting heart rate variation under different experimental conditions is demonstrated below.

### Heart rate adapts to embryo growth and morphology

**Figure 3:**
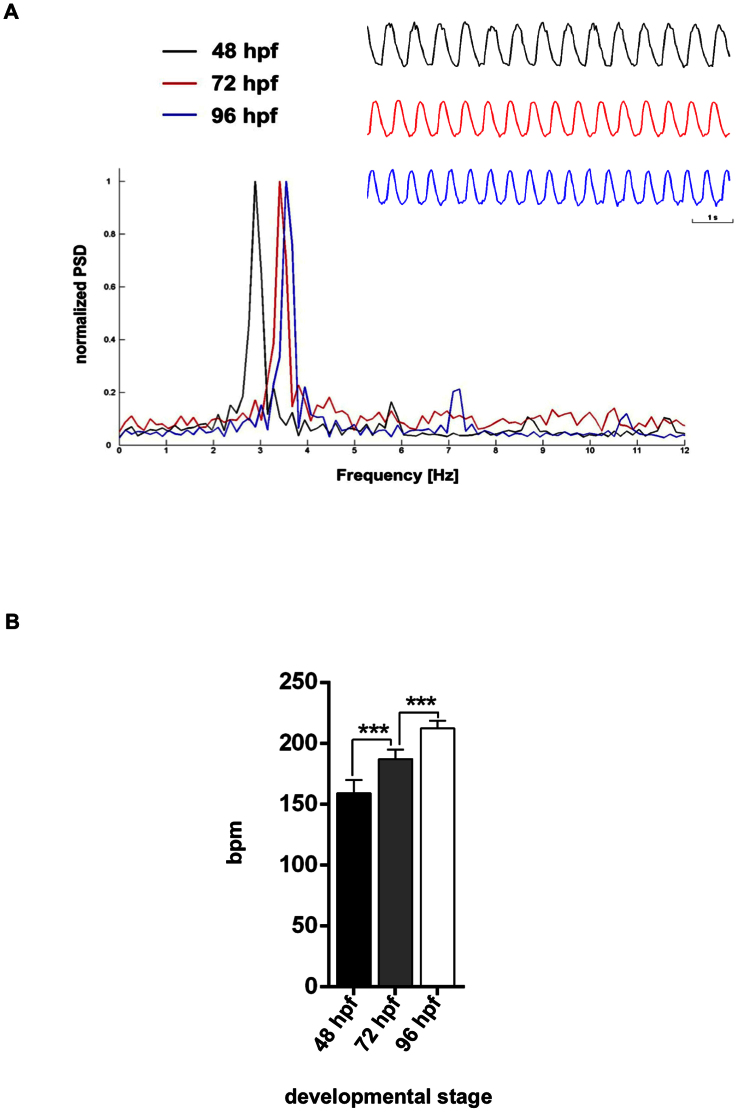
Measurements of increasing heart rate in developing embryos. (A) Representative frequency spectrum of *Tg(Kdrl:GFP)^s843^/Tg(gata1:DsRED)^sd2^* embryos at three different stages, 48, 72 and 96 hpf, using mode 1. The chronologies of the embryos are also indicated. (B) Heartbeat of 85 different transgenic embryos *Tg(Kdrl:GFP)^s843^/Tg(gata1:DsRED)^sd2^* at 48, 72 and 96 hpf, evaluated using ZebraBeat. The average beats per minute (bpm) is shown. Data represent the mean ± SD. The cardiac rate measured by the image-based method increases with the developmental stage, as previously reported^33^.

The embryonic zebrafish heart exhibits the same pattern of electrical excitation as the human heart, with pulses generated in the sino-atrial node, propagated through the atrium, pausing in the atrio-ventricular node, and then sent to the ventricle^14^,^31^. In humans, the normal resting heart rate is between 60 and 90 beats per minute. In many of the more traditional models, the heart rate is markedly different: for instance, the heart rate of a mouse is 300–600 beats per minute. In zebrafish, the normal embryonic heart rate is much closer to that of humans, at 120–180 beats per minute^32^. Zebrafish heart contraction increases as embryonic development progresses to ensure the perfusion of all tissues of the growing embryo^12^. We applied the blood pool method to zebrafish embryos under normal conditions (28°C) at different developmental stages. As an example, Figure 3A shows the frequency spectra of the variation of area over time of 48, 72 and 96 hpf zebrafish embryos. The increase in the cardiac rate of the embryos with gestational age (a shift of the dominant frequency toward the right) can be observed. The cardiac heart rate of 85 zebrafish embryos was measured at 48, 72 and 96 hpf, and the statistical analyses are shown in Figure 3B. In accord with previous results, our findings show a significant increase in the number of beats per minute (bpm) across embryonic development^33^.

These data support the importance of using a standardized method able to detect small changes in heart contractility (of the order of tens of beats per minute) that are otherwise undetectable to the human eye.

### Muscle relaxants and adrenergic receptor-agonists influence heart rate

**Figure 4:**
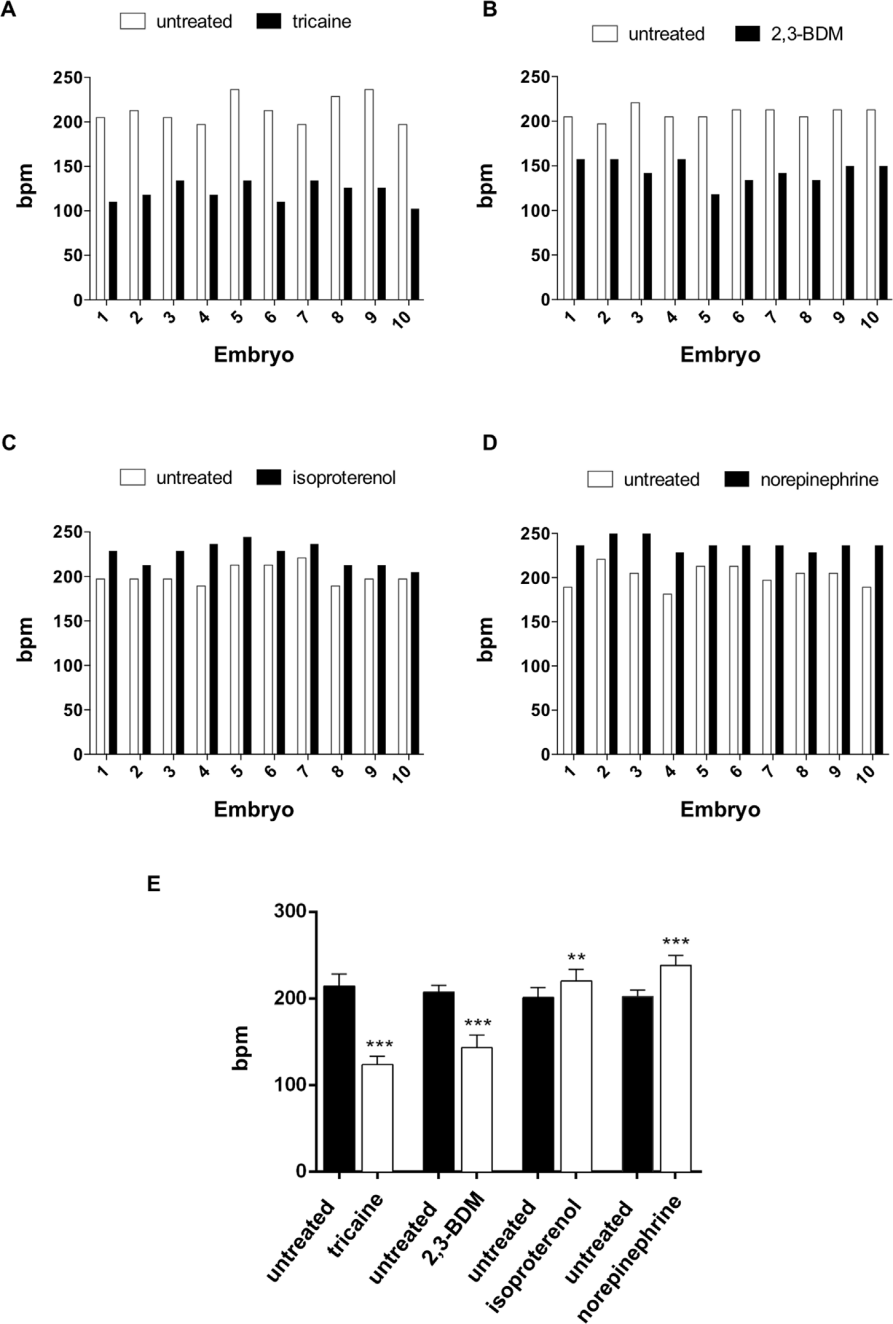
Detection of heart rate variation in zebrafish embryos after treatment with drugs that slow or increase heart contraction rate. Cardiac rate of *Tg(kdrl:GFP)^s843^;Tg(gata1:DsRED)^sd2^* embryos at 96 hpf before and after the administration of drugs that decrease (tricaine in A and 2,3-BDM in B) or increase (isoproterenol in C and norepinephrine in D) zebrafish heart contraction rates. The cardiac rate of 10 embryos was measured immediately before drug administration. Note that the same response to drug administration was observed in all zebrafish embryos. (E) Statistical analysis of 48 embryos indicates that tricaine strongly affects cardiac rate, decreasing the contraction rate up to 43%, whereas administering 2,3-BDM reduced the cardiac rate by approximately 31%. In contrast, administering isoproterenol and norepinephrine induced an increase in cardiac rate of approximately 12% and 18%, respectively. Each histogram represents the mean value of the beat of 48 embryos and shows the mean ± SD.

To demonstrate the effectiveness of the platform under a range of conditions that mimic physiological or pathological cardiac rate variation, four compounds were used to reduce or increase the heart rate^22^,^34^,^35^. The muscle relaxants tricaine and 2,3-BDM or the adrenergic receptor agonists isoproterenol and norepinephrine were administered to pools of ten embryonic zebrafish at 96 hpf, and the cardiac rate was then detected using *ZebraBeat*. As previously published^35^, tricaine and 2,3-BDM reduced the cardiac rate of zebrafish embryos (Figure 4A and 4B, respectively). In contrast, isoproterenol and norepinephrine treatments increased the cardiac rate (Figure 4C and 4D, respectively)^22^. These results were extracted from blood pool chronologies and are summarized in Figure 4E. Tricaine treatment markedly affected the heart rate by reducing it of 43% on average with respect to the pre-administration stage (p < 0.001), whereas the administration of 2,3-BDM led to a 31% reduction in cardiac rate (p < 0.001). In contrast, the administration of isoproterenol induced a moderate increase (12%, on average) in the cardiac rate (p < 0.01). Finally, our analysis showed that the administration of norepinephrine stimulated an 18% increase in the cardiac rate (p < 0.001).

Altogether, these findings confirm that our method rapidly and reproducibly detects small and previously undetected differences in the cardiac contraction rate; thus, it might be suitable for measuring the heart rhythm of embryonic zebrafish under physiological conditions (development) and pathological conditions (genetically or pharmacologically induced alterations).

### Gene silencing might have different effects on cardiac rate

**Figure 5:**
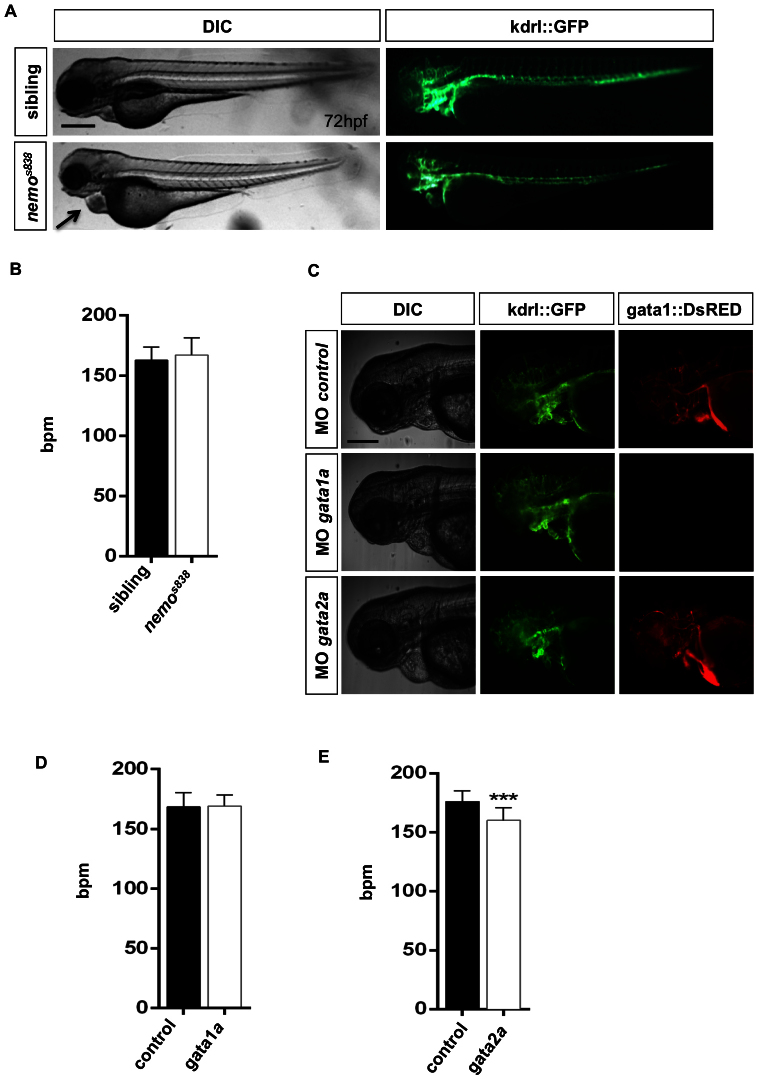
Automated evaluation of gene function *vs* heart rate in zebrafish embryos. (A) Bright-field (DIC) and epifluorescent (GFP) micrographs of *Tg(kdrl:GFP)^s843^* siblings and *nemo* (*nem)^s838^* mutant embryos at 72 hpf. *nemo* mutant embryos displayed dilation of the heart chambers and massive accumulation of erythrocytes in the heart (arrowheads). Scale bar represents 250 μm. (B) Histograms represent the cardiac rate of the siblings compared to *nemo* (*nem)^s838^* mutant embryos using mode 2. The heart rate of *nemo* mutant embryos is comparable to that of the siblings. Each histogram represents the mean value of the heartbeat of 25 embryos and shows mean ± SD. (C) Bright-field (DIC) and epifluorescent (GFP and DsRED) micrographs of *Tg(kdrl:GFP)^s843^/Tg(gata1:DsRED)^sd2^* embryos at 72 hpf that were injected with control, *gata1a* or *gata2a* morpholinos. Note the normal development of the vasculature in all morphants and the absence of red blood cells in *gata1a* morpholino-injected embryos. Scale bar represents 250 μm. (D) Histograms represent the heart rate of *gata1a* morphants compared with control-injected embryos. No variation in heart rate was observed using mode 2 in embryos depleted of *gata1a* expression. (E) Histograms represent the heart rate of *gata2a* morphants compared with control-injected embryos. Silencing of *gata2a* expression significantly reduced the heart rate of these morphants. Each histogram represents the mean value of the heartbeat of 32 embryos and shows the mean ± SD.

A number of zebrafish mutants presenting defects in heart morphology have been identified in recent years^11^,^12^,^14^,^36^. Most of these mutants were positionally cloned, and new genes affecting heart development and function were identified. In a recent ENU-based mutagenesis screen, we identified the *nemo (nem)^S838^* mutant^27^. *nemo* shows a progressive reduction in blood circulation starting from 24 hpf, and circulation is completely absent after 48 hpf, when red blood cells accumulate at the level of the inflow tract (Supplementary Movie 4). Despite this phenotype, *nemo (nem)^S838^* embryos grow normally and display a normal patterning of the vasculature (Figure 5A), which starts to regress at 72 hpf. At this stage, *nemo* *(nem)^S838^* embryos shows prominent cardiac edema and a marked accumulation of blood in both cardiac chambers, which become dramatically enlarged and have impaired blood flow. In this study, we used the area variation mode to monitor the cardiac rate of *nemo (nem)^S838^* mutants. The method was used to monitor the *nemo (nem)^S838^* cardiac rate at 48 hpf to avoid measuring the heart rhythm of definitively functionally compromised hearts. Although some arrhythmias were observed during the imaging of *nemo* *(nem)^S838^* embryo hearts, no marked alteration of cardiac rhythm was found compared to wild-type control embryos (Figure 5B). These findings indicate that our method can be a useful and affordable instrument for establishing the cardiac function of a gene.

As proof of concept that *ZebraBeat* can be applied to investigate gene-heart associations, we also analyzed the role of two hematopoietic genes, *gata1a* and *gata2a*, in the cardiac function embryonic zebrafish in terms of variation of the cardiac rhythm. The choice of *gata1a* and *gata2a* morphants was driven by the fact that the loss of *gata1a*, but not *gata2*a, has been shown to induce the conversion of erythropoiesis to myelopoiesis, with the consequence that embryos lacking *gata1a* do not have erythrocytes^29^. Conversely, in *gata2a* morphants, fewer circulating red blood cells were observed. It was also observed that both *gata1a* and *gata2a* morphants have normal heart chamber patterning and vascular development^30^.

Owing to the absence or reduced number of circulating red blood cells in *gata1a* and *gata2a* morphants, respectively, we applied the area variation approach to quantify the cardiac rate in these morphants. This approach is applicable when an alteration in the total number of erythrocytes occurs as a result of genetic modifications or the circulation is interrupted (see the *nemo* analyses as an example). Using morpholino injection, we evaluated the implications of blocking the function of these two genes on cardiac rate. Measurements were performed in control embryos and *gata1a* and *gata2a* morphants at 72 hpf (Figure 5C). The automated monitoring of the cardiac rate confirmed that in *gata1a* morphants, even in the absence of circulating red blood cells, the embryonic heartbeats at a regular rate, comparable to controls (Figure 5D). Interestingly, *gata2a* morphants, although not presenting evident alterations of heart morphology, showed a reduction in the cardiac rate compared with control embryos (Figure 5E). These data are in agreement with the critical role played by the intracardiac reversal of blood flow in valve morphogenesis^30^. Because *gata1a* morphants exhibit an increased reversed flow, whereas this is reduced in *gata2a* morphants, the cardiac rate reduction observed in *gata2* morphants is most likely due to the abnormal valve expansion induced by the reduced reverse flow. Finally, we conclude that the circulation of red blood cells is not essential for proper heart development, morphology and contraction rate.

Our findings support the need for effective tools to investigate and discover unexpected functions of genes in cardiac contractility during early developmental stages.

### Pharmacological treatment modulates heart activity

The zebrafish is a key model organism for drug screenings^4^,^29^,^37^,^38^. In this study, we investigated the role of various small molecules with cardiovascular-related function on the cardiac rate of zebrafish embryos. We selected compounds that do not markedly affect heart development in embryonic zebrafish (Figure S2). The aim was to test the strength of our method for automated cardiac rate monitoring during high-throughput drug screening.

**Figure 6:**
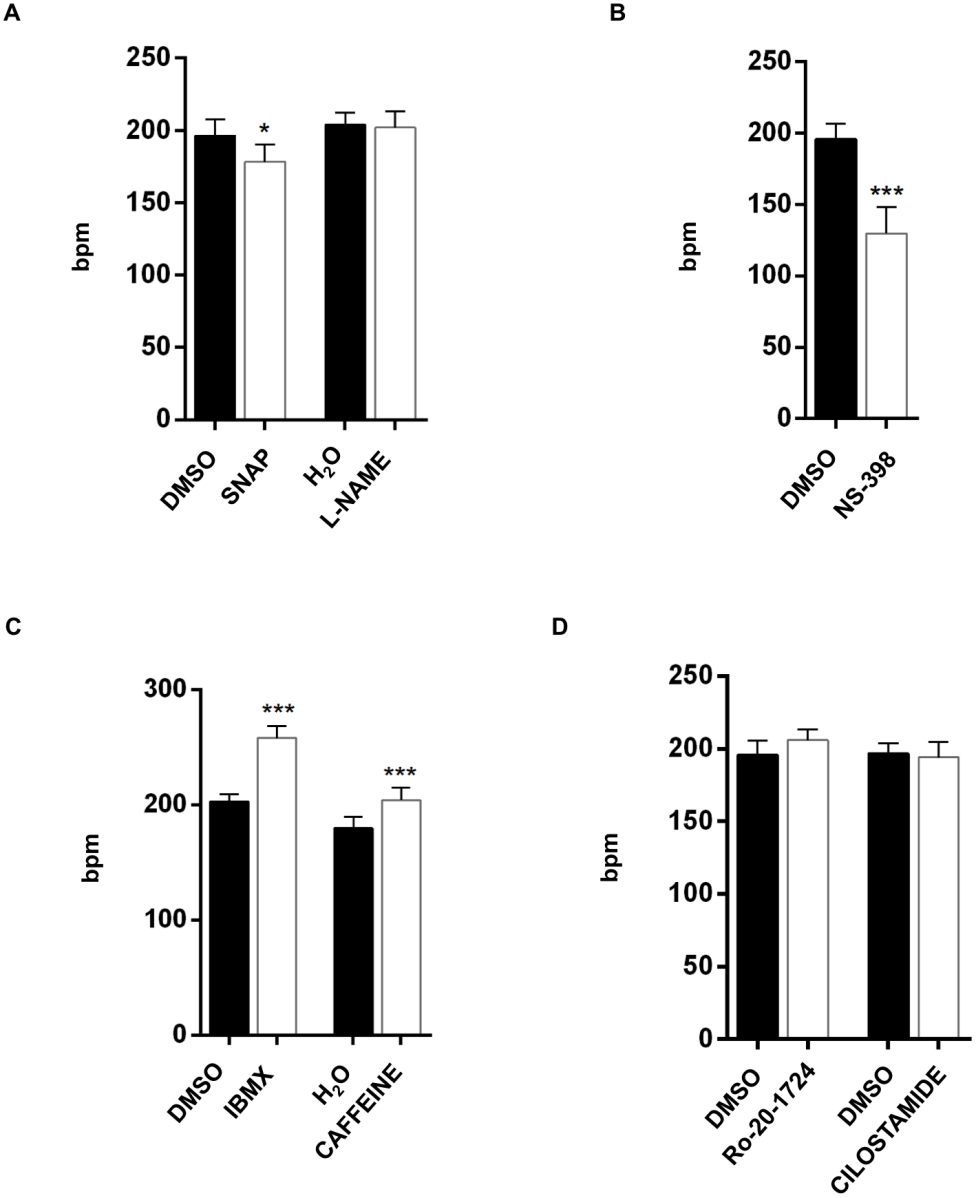
Treatment with cardiovascular-related drugs causes significant alterations in the heart rate of embryonic zebrafish. (A) Treatment of *Tg(kdrl:GFP)^s843^;Tg(gata1:DsRED)^sd2^* with the NO donor molecule SNAP induced a slight but significant reduction in heart rate compared to treatment with the vehicle DMSO at 72 hpf. In contrast, L-NAME administration did not affect zebrafish heart rate. (B) Addition of the COX2 inhibitor NS-398 induced a significant reduction in heart contraction compared with control treatment. (C) The pan PDE inhibitors IBMX and caffeine increased zebrafish heart rate, whereas cilostamide, a selective inhibitor of PDE3, or Ro-20-1724, a selective inhibitor of PDE4, did not affect heart rate. Each histogram represents the mean value of the heartbeat of 32 embryos for each treatment and shows the mean ± SD. The results were obtained by applying the area variation method in this case, but results from the blood pool method are identical.

Nitric oxide (NO) is a short-lived molecule produced by almost all cell types in the myocardium; it regulates cardiac function through both vascular-dependent and -independent effects^39^,^40^,^41^. NO modulates inotropic and metabolic responses that initiate cGMP signaling through the activation of soluble guanylyl cyclase (sGC)^42^. To investigate the effects of NO on the cardiac performance of zebrafish embryos, we focused our attention on variations in the measured cardiac rate upon administration of SNAP, a vasodilatory molecule that supplies NO (“NO donor”)^43^. Furthermore, we measured the cardiac rate after administration of the NOS enzyme inhibitor L-NAME, a vasoconstrictor drug that decreases the production of NO. As shown in Figure 6A, exposure to SNAP provoked a reduction in the cardiac rate compared to control-treated embryos, whereas the administration of L-NAME did not alter the cardiac rate.

Recent findings shed light on the role of cyclooxygenase 2 (COX-2), an inducible enzyme that mediates the generation of prostaglandins during inflammation, in heart function and pathology^44^. We analyzed the effects of treating embryos with the COX2 antagonist NS-398. Remarkably, we observed that NS-398 administration induced a significant decrease in the cardiac rate without affecting heart morphology (Figure 6B).

Another important player for regulating cardiac muscle contraction is cAMP-dependent signaling. In myocardial cells, the increase in concentration of the second messenger cAMP may lead to the activation of cyclic nucleotide-gated ion channels, thereby, inducing the increase of heart contraction^45^. The reduction of cellular cAMP levels is mediated by the activity of phosphodiesterases, which dephosphorylate cAMP into AMP. To investigate the effects of PDE inhibition on embryonic heart contraction, we treated embryos with non-selective PDE inhibitors, IBMX and caffeine. These drugs induced an increase in the heart rate (Figure 6C). Because phosphodiesterases 3 (PDE3) and 4 (PDE4) are the main regulators of cardiac muscle contraction, accounting for more than 90% of specific activity for cAMP hydrolysis in animal hearts^46^, we used cilostamide, a selective inhibitor of PDE3, and Ro-20-1724, a selective inhibitor of PDE4, in our tests. We did not detect any variation in the cardiac rate (Figure 6D) as a consequence of the administration of these drugs. These results are in contrast to the cardiac rate increase observed when pan-PDE inhibitors were administered. However, this finding can be explained by the lack of specificity of these pan inhibitors for the zebrafish PDE isoforms.

These results confirm the utility of our automated tool for detecting variations in heart rhythm induced by chemical treatments.

### An image-based platform suitable for investigating the coupling of ventricular and atrial contraction

**Figure 7:**
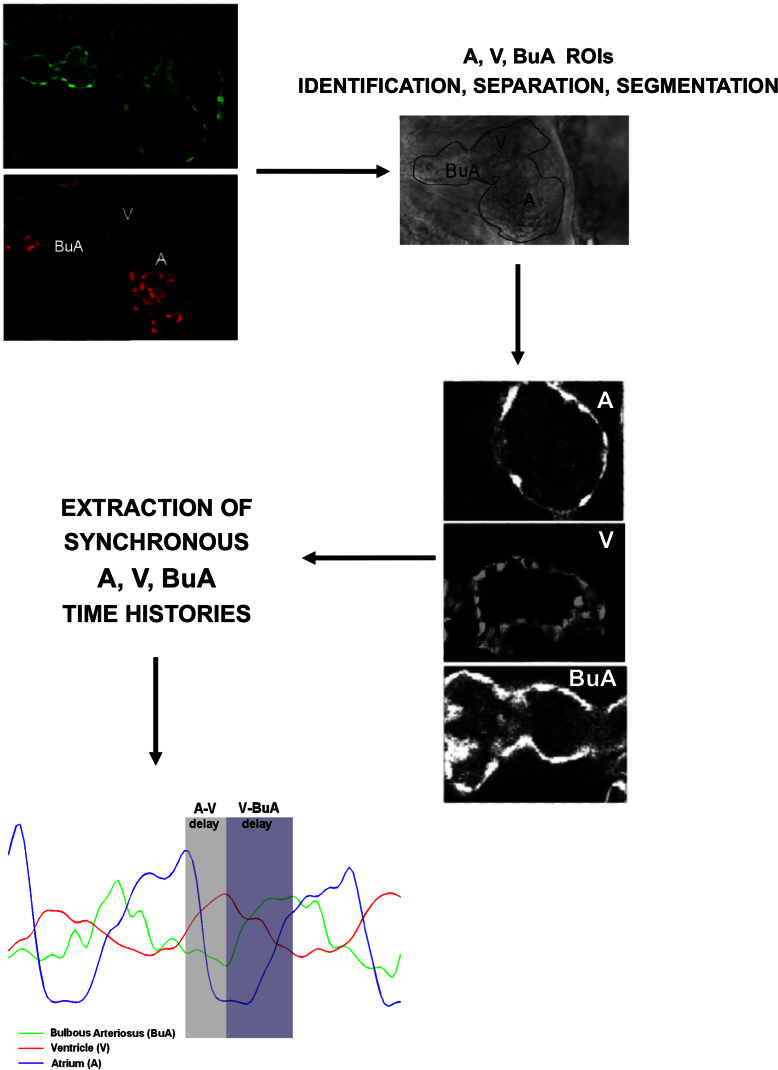
Synchronicity of cardiac compartments in the embryonic zebrafish heart. Analysis of atrium, ventricle and bulbus arteriosus synchronicity in cardiac chambers of 96 hpf embryonic zebrafish. After acquisition, we manually identified and selected three ROIs, corresponding to the atrium (A), the ventricle (V) and the bulbous arteriosus (BuA). Three chronologies were then extracted and analyzed, allowing a quantitative analysis of the level of A, V and BuA coupling contractions. These data were obtained by applying mode 1, showing that the blood motion from A (beforehand peak value, with respect to ventricular peak value) to V and then to BuA (delayed peak value, with respect to ventricular peak value) can be depicted and that A-V and V-BuA delays can be quantitatively assessed.

The image-based platform presented here is also suitable for monitoring all three cardiac compartments (the atrium, ventricle and bulbous arteriosus) from the same image acquisition set and for quantitatively analyzing the synchronicity of these chambers. We present an analysis of the synchronicity of the cardiac chambers of a wild-type 96 hpf embryonic zebrafish (Figure 7). As shown in Figure 1B, we manually identified and selected three ROIs, corresponding to the atrium, ventricle and bulbous arteriosus. Over each selected ROI, the same automated analysis was performed (in both mode 1 and mode 2). The chronologies of each region allowed a quantitative analysis at the level of the coupling of ventricular, atrial and bulbous arteriosus contraction. This was easily performed by evaluating the time delay between the peak values of the extracted synchronous chronologies within the same cardiac cycle. Figure 7 clearly shows that this method allows depiction of blood motion from the atrium (beforehand peak value, with respect to ventricular peak value) to the ventricle and then to the bulbous arteriosus (delayed peak value, with respect to ventricular peak value) before being distributed to the peripheral circulation.

In summary, this approach is very helpful for investigating the uncoupling or delayed coupling of ventricular and atrial contraction (e.g., during atrio-ventricular blockade) in the zebrafish animal model.

## Discussion

The zebrafish heart, with its similarity to the human heart, is a unique system for modeling both genetic and drug-driven heart failure conditions^11^,^12^. Additionally, the zebrafish model has generated data with important implications in the fields of evolutionary, developmental and regenerative biology and potentially offers insight into pathways that lead to congenital heart disease in children^47^,^48^. Therefore, unabated effort must be devoted to this animal model to continue its use for therapeutic studies. In this study, we described an image-based method for measuring the cardiac rate of zebrafish embryos. The method and its implementation take advantage of a combination of transgenic fluorescent zebrafish lines (*Tg(kdrl:GFP)^s843^; Tg(gata1:DsRED)^sd2^*, a confocal microscope with a resonant scanner, and a flexible algorithm that extracts information from sequential confocal images and yields the cardiac rate of the analyzed animals. The flexibility of our approach allows the proper selection of a strategy for image processing, which also makes it possible to use the software for analyzing animal models in which an alteration in the total number of erythrocytes or the interruption of the circulation occurs as a result of genetic modifications. This can be easily done by simply selecting the area variation or the blood pool variation scheme for image analysis in the software graphical user interface.

Our method offers several advantages because it is non-invasive, quantitative, simple and operator independent. In fact, the analysis is fully automated and allows the determination of the number of heart contractions per unit of time for zebrafish embryos without the need for expert operators. Moreover, it allows the analysis of the whole pool of embryos positioned in a multiwell plate as a single operation, thus making the approach high-throughput.

Previous studies have proposed similar approaches based on the integration of image processing and power spectral analysis to evaluate the embryonic zebrafish cardiac rate^16^,^19^. The approach proposed by Chan et al. estimates the cardiac rate from images of the blood pool in the caudal vasculature of the embryonic zebrafish. With respect to the method proposed here, the approach of Chan and colleagues suffers from some limitations, such as the need for caudal vein imaging at a fully mature and developed stage (the vein region usually undergoes specific reorganization for the first 72 h after fertilization). Therefore, Chan and colleagues method cannot be used for heartbeat measurement in young zebrafish embryos (24 to 72 hpf embryos). Moreover, the method proposed by Chan and colleagues is not suitable for quantifying the cardiac rate when the total number of erythrocytes is altered or the circulation is interrupted as a result of genetic modifications. By contrast, Milan and colleagues previously proposed a different method^22^. The limitations of that method are the need to perform a power spectral analysis of embryonic ventricle motion (using ventricular rate as the index of heart rate) to determine the cardiac rate of the animals: movement from all of the tissues around the heart can interfere with this analysis, with the consequence of a high signal-to-noise ratio. In contrast to the method proposed by Milan et al.^22^, the segmentation strategy is the basis for the area variation method proposed in this manuscript to limit the artifacts that may arise from the movement of tissues around the heart, improving the quality of the signal. As for the approach proposed by Schwerte et al.^16^, the luminance profile of single lines drawn crossing the central ventricle and atrium should be dependent on blood pool movement into the chambers, and for this reason, this method is not suitable for embryos with impaired blood cell development or lack of blood flow. Moreover, the method proposed by Schwerte is not fully automated^16^.

The main advantages of the method proposed here are due to the implementation of two image processing strategies (modes), which allows cardiac rate evaluation of embryos with a reduced number of blood cells or lacking blood circulation, which may occur with specific genetic modifications or drug treatments. In such situations, the user can switch from the blood pool mode (based on blood cell counting) to the area variation mode (based on segmentation of the cardiac chamber walls) to analyze the same dataset of acquired images. This switch can be performed with negligible computational effort in terms of time for processing images. We demonstrated such efficacy by analyzing embryos with genetic mutations that cause the suppression of hematopoietic gene expression, which leads to a lack of red blood cells or the reduction and in some cases even the interruption of blood circulation.

A possible limitation of the method is the need for a fluorescent reporter line in which the heart chambers and/or the blood cells are marked with different fluorescent markers (e.g., GFP or cherry). However, these Tg lines are now widely used in all zebrafish research laboratories worldwide. Another possible limitation is represented by the prototype version of the software. However, future releases of the software will be easily moved to open-source platforms that will (1) allow further improvements to its performance in terms of computational time and will (2) help to make the software available without licensing costs.

The zebrafish is an established model for in vivo drug discovery^17^,^49^,^50^,^51^ and large forward genetic screens^27^,^52^,^53^. Recently, systematic approaches to toxicology studies have been performed using the zebrafish model^54^,^55^,^56^. Much effort is being made to develop high-throughput imaging platforms for the application of zebrafish in large-scale in vivo drug and toxicological screenings^57^,^58^,^59^. Although screening of drug candidates affecting heart development or with cardiotoxicity has been widely performed in zebrafish^59^,^60^,^61^,^62^, the identification of specific drugs or small molecules affecting cardiac rate and function remains an unexplored field^63^. The lack of this type of study is due mainly to the absence of a reliable automated tool for identifying cardiac parameters, such as frequency or fractional shortening, after drug treatment or due to genetic mutations. The method proposed here and its implementation appear to be sensitive to pharmacological and genetic interventions and provide a relatively inexpensive tool with which to characterize subtle changes in cardiac function that result from genetic and pharmacological manipulation. Therefore, the utility of this method resides in its suitability for monitoring variations in cardiac rate during high-throughput drug screening and pharmacological testing in a cost-effective and highly reproducible manner. The identification of new mutants with subtle cardiac alterations (resembling pathological conditions in humans) by using large forward genetic screens can also be achieved. This platform is potentially adaptable to an array of different biological models, ranging from Drosophila adults to mouse embryos.

In summary, the combination of this method (and its associated software) with a resonant confocal microscope could allow laboratories to perform accurate, automated, reliable and high-throughput measurements of cardiac rate in developing zebrafish embryos under normal and pathological conditions.

## Supplementary Material

Supplementary Information Supplementary figures

Supplementary Information video s1

Supplementary Information video s2

Supplementary Information video s3

Supplementary Information video s4a

Supplementary Information video s4b

Supplementary Information video s4c
