# Supplementary material for: ZebraBeat: a flexible platform for the analysis of the cardiac rate in zebrafish embryos
Source: Sci Rep. 2014 May 9;4:4898. doi: 10.1038/srep04898 (PMC4790192; doi:10.1038/srep04898)
Supplement: Supplementary Information — Supplementary figures [file srep04898-s1.pdf]

ZEBRABEAT: A FLEXIBLE PLATFORM FOR THE ANALYSIS OF THE CARDIAC RATE IN ZEBRAFISH EMBRYOS

Elisa De Luca[1], Gian Maria Zaccaria [2], Marwa Hadhoud [3],Giovanna Rizzo[4], Raffaele Ponzini [5], Umberto Morbiducci[2] and Massimo Mattia Santoro [1]

[1] Department of Molecular Biotechnology and Health Sciences and Molecular Biotechnology Center, University of Torino, Turin, Italy;
[2] Department of Mechanical and Aerospace Engineering, Politecnico di Torino, Turin, Italy;
[3] Faculty of Engineering, Helwan University, Helwan, Egypt
[4] Institute of Bioimaging and Molecular Physiology, CNR, Milan, Italy;
[5] SuperComputing Applications and Innovation Department, CINECA, Milan, Italy

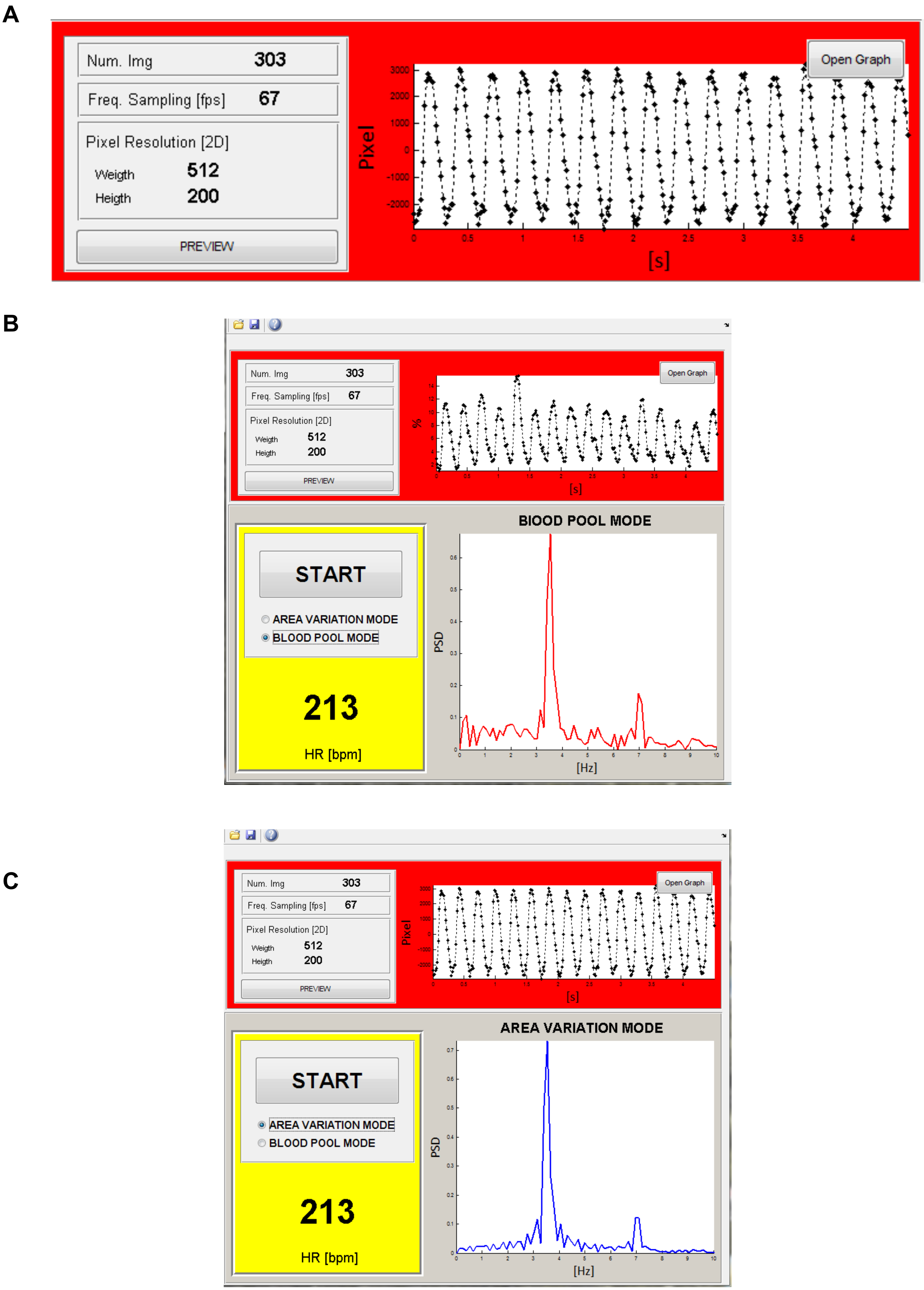
A
Num. Img 303
Freq. Sampling [fps] 67
Pixel Resolution [2D]
Weigth 512
Heigth 200
PREVIEW
Open Graph
Pixel
[s]
B
BIOOD POOL MODE
START
AREA VARIATION MODE
BLOOD POOL MODE
213
HR [bpm]
PSD
[Hz]
%
C
AREA VARIATION MODE

Figure S1

Supplementary Figure S1. *ZebraBeat* graphical user interface I.

Explicative snapshots of the *ZebraBeat* graphical user interface. The graphical interface allows users to (1) automatically load images acquired from confocal microscope and (2) check for image quality by visual inspection (PREVIEW button, panel A), before starting the image processing step (START button).
Depending on the purpose of the experiments and/or the type of dataset that need to be investigated, the user can select the “blood pool” mode 1 (B) or “area variation” mode 2 (C). In the first case, only red channel images will be automatically selected and processed by the software. In the second case only green channel images will be automatically selected and processed by the software. As a result of the selected image processing strategy, the frequency spectrum (Hz) and the numeric value of the extracted zebrafish cardiac rate (bpm) will be visualized on the screen. Here, we show measurement of zebrafish embryo heart rate where both blood cells and heart wall are stained by two different fluorescent protein (GFP) and (DsRED). Both modes (blood pool and area variation) working independently on the same set of images (*Tg(kdrl:GFP)*^{s843}*;Tg(gata1:DsRED)*^{sd2} embryos at 48 hpf) have yield the same value of heart rate and frequency indicating the bona fide and mutually exclusive function of modes (C).

A

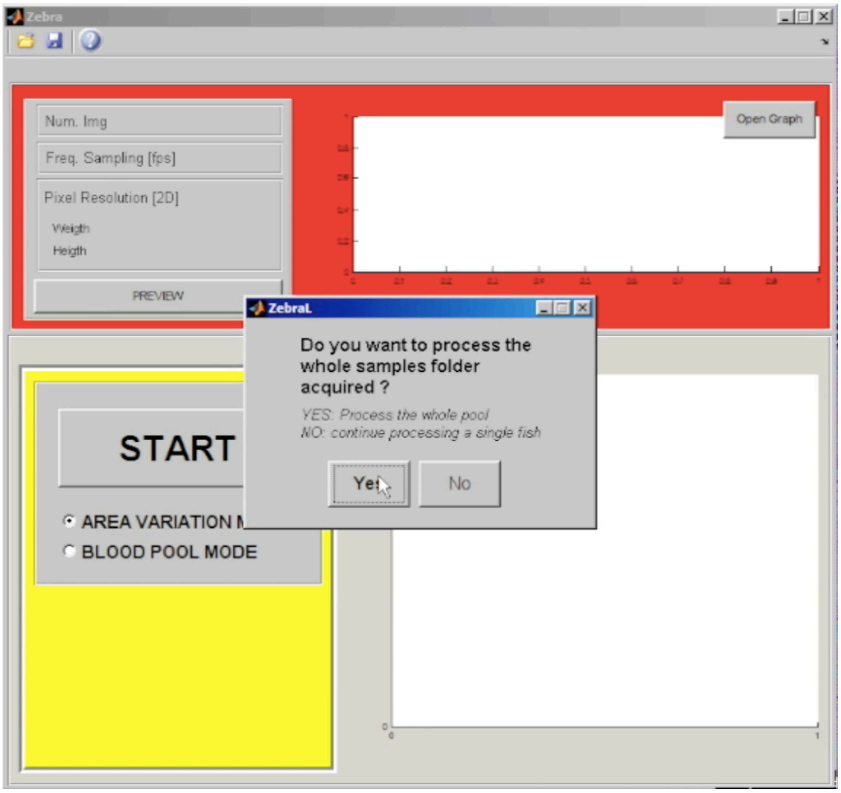

B

	AREA VARIATION MODE	BLOOD_POOL_MODE
1	189	189
2	205	205
3	197	197
4	213	213
5	221	221
6	110	110
7	205	205
8	189	189
9	213	213
10	213	213
11	197	197
12	229	229
13	213	213
14	205	205
15	213	213
16	213	213
17	236	236
18	205	205
19	229	229
20	213	213

Figure S2

Supplementary Figure S2. *ZebraBeat* graphical user interface II. Explicative snapshots of the *ZebraBeat* graphical user interface. The user interface allows users to easily automatically analyze images (1) of a single embryo or (2) of the whole pool of acquired embryos that are present into the multiwell plate (panel A). If the operator makes the choice for analyzing all the acquired embryos, a report is generated containing the cardiac rate of all the embryos and their identifier (panel B). After having analyzed the whole dataset, the operator can always move back to the time history and/or the frequency spectrum of a specific embryo of the analyzed pool by simply loading, using the graphical interface, the stored subfolder containing the analyzed images of the embryo of interest (as in Figure S1).

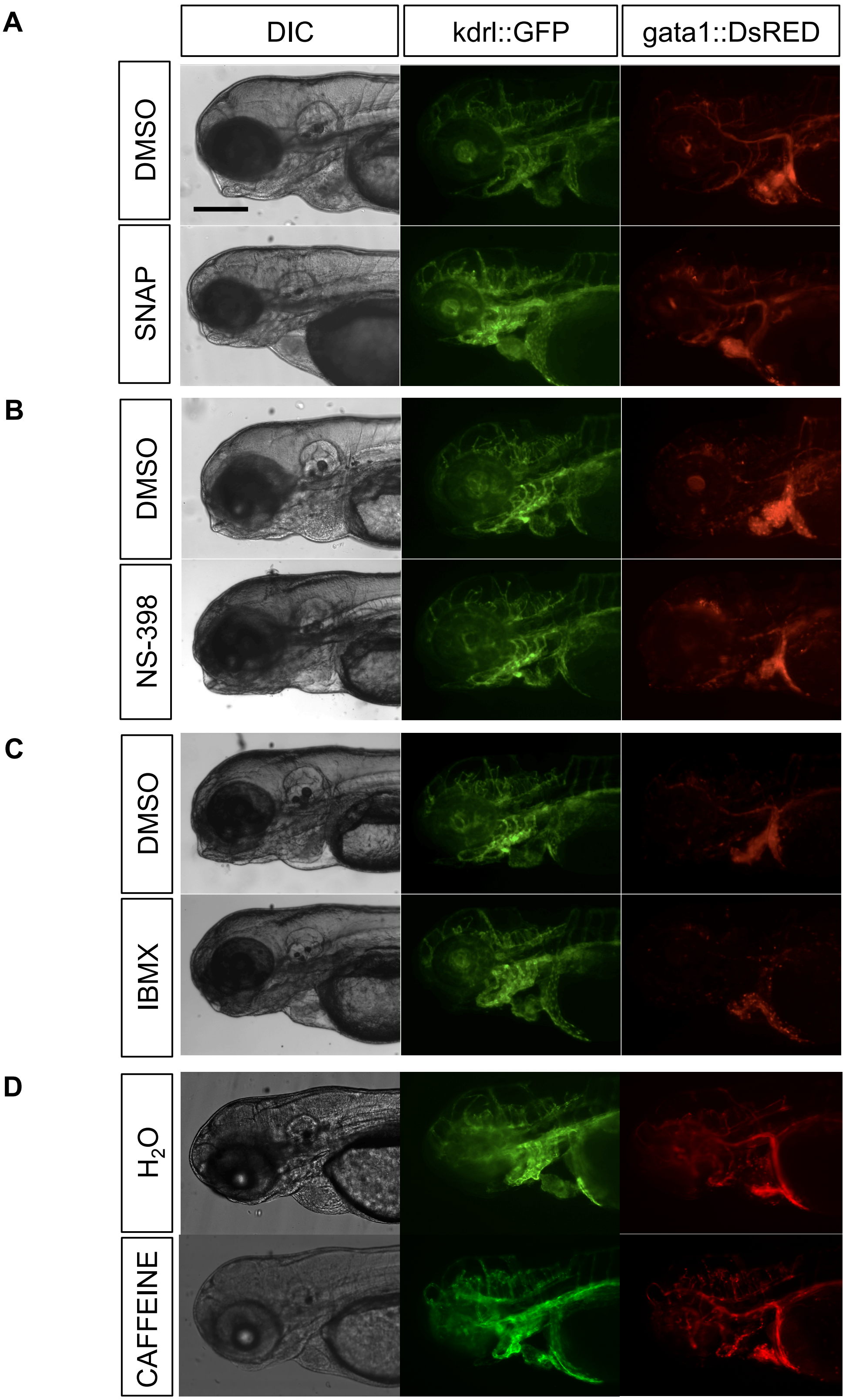
A
B
C
D
DIC
kdrl::GFP
gata1::DsRED
DMSO
SNAP
DMSO
NS-398
DMSO
IBMX
H2O
CAFFEINE

Figure S3

Supplementary Figure S3. Morphological effects of cardiovascular-related drugs on zebrafish embryos.

Representative images of *Tg(kdrl:GFP)^{s843};Tg(gata1:DsRED)^{sd2}* embryos at 72 hpf treated with molecules that are related with cardiovascular functions in vertebrates. The association of these drugs with the modulation of zebrafish cardiac contraction has not yet been reported in zebrafish cardiac development. Administration of (A) SNAP, (B) NS-398, (C) IBMX, and (D) caffeine does not induce any visible alteration of the cardiac morphology when compared with the corresponding vehicle alone. Scale bar represents 250 μm.

Supplementary Movie 1. Representative movie of double transgenic embryo *Tg(kdrl:GFP)^{s843};Tg(gata1:DsRED)^{sd2}* at 96hpf.
A dual color fluorescent movie of a living 96hpf transgenic embryo *Tg(kdrl:GFP)^{s843};Tg(gata1:DsRED)^{sd2}* has been acquired with the Leica MZ16.5 fluorescent stereomicroscope and process with Zeiss AxioVision software. This movie shows the vascular network (endothelial cell in green) and the blood flow (blood cells in red).

Supplementary Movie 2. Visualization of heart beating in live zebrafish transgenic embryo *Tg(kdrl:GFP)^{s843};Tg(gata1:DsRED)^{sd2}* at 96hpf by confocal resonant scanner.
Dual color fluorescent movie of the beating heart of 96hpf transgenic embryo *Tg(kdrl:GFP)^{s843};Tg(gata1:DsRED)^{sd2}* acquired with the resonant scanning confocal microscope and shown at the temporal resolution 5fps.

Supplementary Movie 3. Demonstration of the user-friendly interface of the *ZebraBeat* software. Step 1: user can load the whole pool acquired (n samples) – YES, or just loads one single sample – NO. Step 2A: software automatically saves the table that includes all HR samples. Step 2B: user can start the software and select the mode accordingly. Step 3B: user can save the results in a separate new file.

Supplementary Movie 4. Representative movie of *nemo* mutant in double transgenic embryo *Tg(kdrl:GFP)^{s843};Tg(gata1:DsRED)^{sd2}* at 48hpf.
(A) Bright-field and (B-C) fluorescent movies of 48hpf *Tg(kdrl:GFP)^{s843}* nemo (nem)^{S838} mutant embryos. Note the absence of circulating blood cells (red channel) and their accumulation in the region of the inflow tract.
